# A Case of Giant Ethmoid Sinus Osteoma

**DOI:** 10.7759/cureus.18011

**Published:** 2021-09-16

**Authors:** Abdullah S Alkhaldi, Shmokh Alsalamah, Tariq Tatwani

**Affiliations:** 1 Medicine, King Saud Bin Abdulaziz University for Health Sciences, College of Medicine, Riyadh, SAU; 2 Otolaryngology, Prince Sultan Military Medical City, Riyadh, SAU

**Keywords:** sinus surgery, paranasal sinus, ethmoid osteoma, ethmoid sinus, osteoma

## Abstract

Paranasal sinus osteomas are slow-growing benign tumors. They are generally asymptomatic in most patients and usually diagnosed incidentally with a sinus radiograph or more frequently with a CT scan of the paranasal sinuses. Osteomas can cause various signs and symptoms, depending on the location of the mass. Giant osteomas of the paranasal sinuses are very rare, with only a handful of case reports in the literature. Due to the rarity of giant osteomas, the clinical presentation and treatment are unclear. In this article, we present a case of giant ethmoid sinus osteoma, which was removed with an endoscopic endonasal approach, as well as a review of the literature.

## Introduction

Paranasal sinus osteomas are slow-growing benign tumors originating from an osteogenic origin [1,2]. These tumors have a 3% prevalence in the general population [3,4]. Osteomas generally occur in the frontal and ethmoid sinuses, with a higher proportion in the frontal sinus [4]. Osteomas are usually asymptomatic and frequently detected incidentally with a paranasal CT scan [2,4]. The tumors occur more in males, aged between 30 to 60 years [2,3]. They are classified as small or giant osteomas, depending on the size of the tumor. Small osteomas, which are more prevalent with a size of less than 3 cm, are usually asymptomatic and require only periodic CT follow-up to assess the size [4,5]. Follow-up focuses on the size of the tumor, to determine growth and the possibility of complications [2,6,7]. Giant osteomas are larger than 3 cm [5] and can cause serious symptoms or complications if they invade adjacent structures. Due to the rarity of giant osteomas, the clinical presentation and treatment are unclear. Giant osteomas of the paranasal sinuses are very rare, with only a few cases published. In this paper, we present a case of giant ethmoid sinus osteoma, which was removed with an endoscopic endonasal approach, in addition to a review of the literature.

## Case presentation

**Figure 1:**
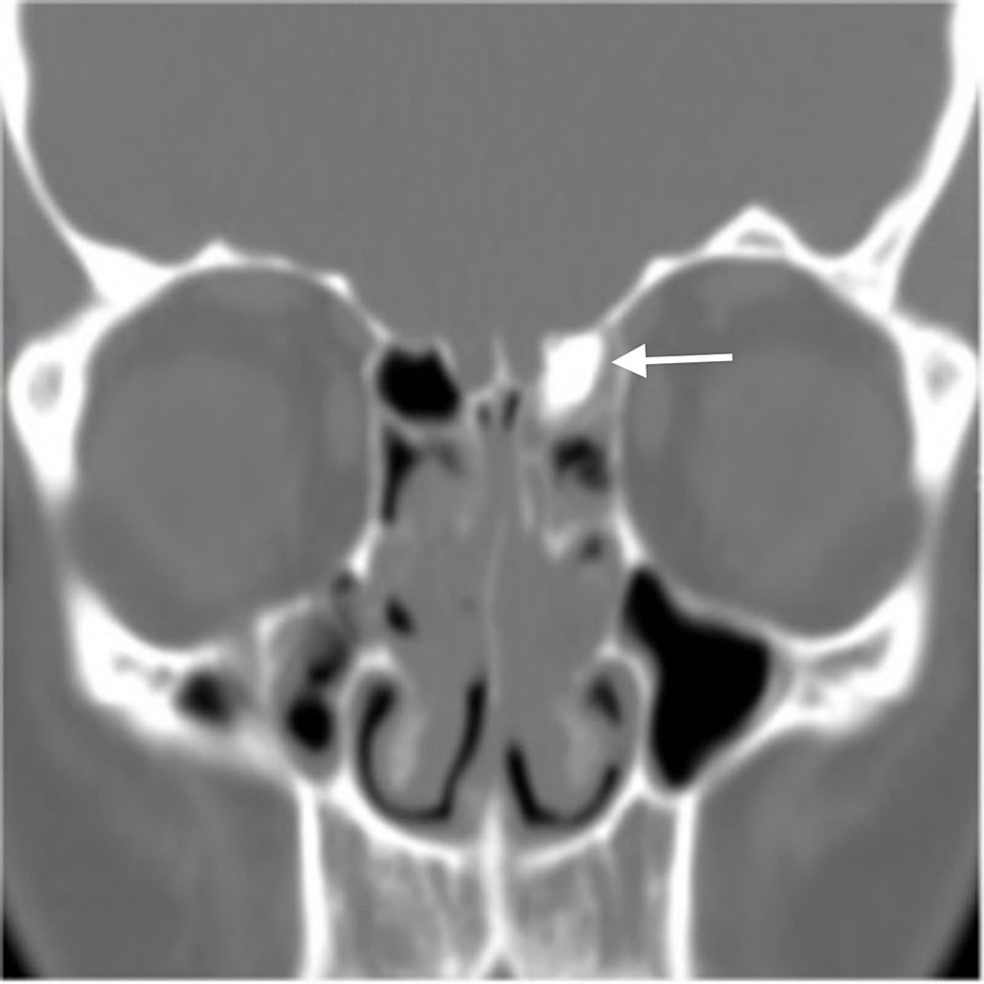
Old CT scan of the patient (performed in 2008): coronal view shows small hyperdense lesion within left ethmoid sinus.

**Figure 2:**
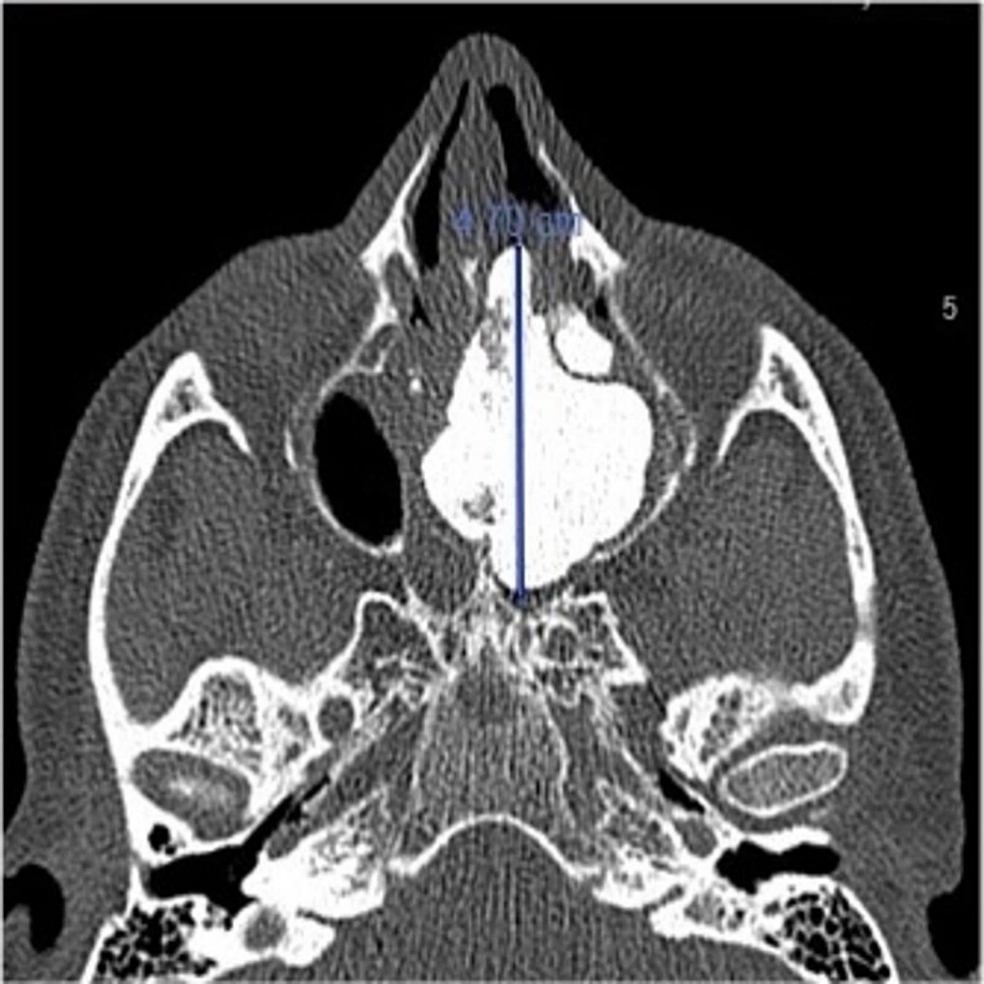
Preoperative CT scan: axial view shows 4.7 x 3.5 cm left ethmoid lobulated hyperdense lesion.

**Figure 3:**
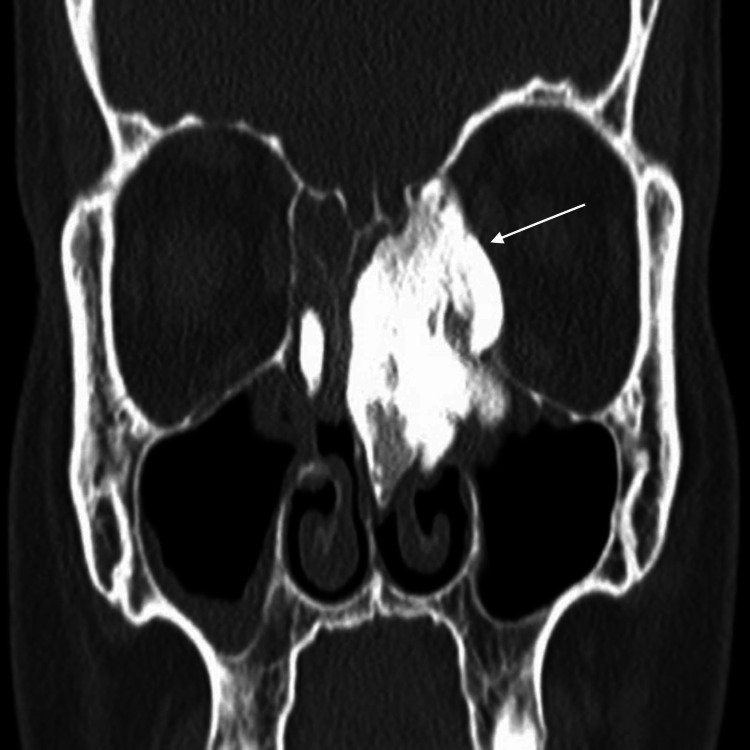
Preoperative CT scan: coronal view shows lesion extension from left ethmoid sinus into left orbital cavity.

**Figure 4:**
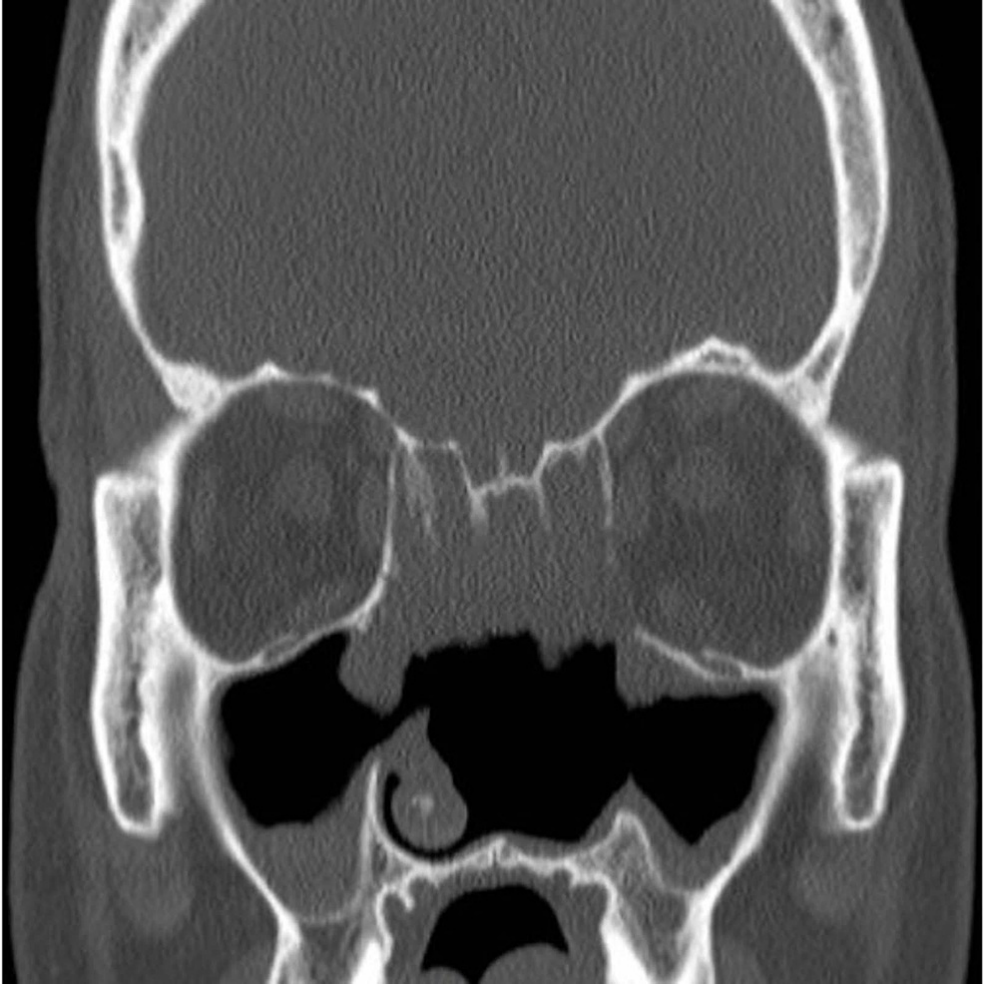
Postoperative CT scan: coronal view shows no residual masses.

A 44-year-old male presented to the otolaryngology clinic with complaints of recurrent symptoms of chronic rhinosinusitis. He was medically free, with no history of allergies or trauma. He had a surgical history of functional endoscopic sinus surgery (FESS) in 2009. On examination, a nasal endoscopy revealed bilateral grade III polyps. An eye examination showed no abnormalities. For further evaluation, a non-contrast CT of the paranasal sinuses was performed and compared with a previous image (Figure 1). The CT scan revealed a large lobulated hyperdense lesion, measuring 4.7 X 3.5 cm, within the left ethmoid sinus. The lesion extended into the left orbital cavity, across the remodeled and eroded lamina papyracae, with encroachment into the ostium of the left maxillary sinus (Figures 2-3). The need for surgical intervention was explained to the patient and he consented to a FESS procedure with the removal of the mass, with or without an external approach. The surgical technique was endoscopic endonasal, without an external approach. Intraoperatively, the examination of the nose showed a huge mass occupying the nasal cavity at the posterior ethmoidal region bilaterally. Using a surgical chisel, the mass was separated from the left orbit. A septectomy was performed to manipulate the mass. The mass was larger than the diameter of the nostril, so a surgical saw was used to fragment the bony mass. Finally, we were able to excise the huge mass in two separate sections. After the surgery, a CT scan showed no residual masses and the patient had an uneventful recovery period (Figure 4). The histopathology report confirmed the diagnosis of a benign ethmoid sinus osteoma. 

## Discussion

Osteomas are the most frequent benign tumors of the paranasal sinuses [8]. Ethmoid sinus osteomas are slow-growing lesions that are generally asymptomatic in most patients and usually diagnosed incidentally with a sinus radiograph or more frequently with a CT scan of the paranasal sinuses [9,10]. 

Osteomas can cause various signs and symptoms, depending on the location of the mass. Headache and facial pain are mostly reported due to sinusitis or the compression of adjacent tissues. If the giant osteoma is close to the orbit or extending intraorbitally, it can cause ocular signs, such as exophthalmos, diplopia, orbital cellulitis, epiphora, and proptosis [11-13]. As in the case of our patient, intraorbital extension is observed in 68.9% of cases [2]. If the osteoma extends intracranially or close to the skull base and is in contact with the dura mater, it can cause serious complications such as meningitis, seizures, or subdural or intracranial abscesses [14,15]. Should the osteoma extend to the nasal passage, nasal obstruction and anosmia are prevalent. Paranasal osteomas can also cause cosmetic deformities and mucoceles [11].

The most frequent sites for paranasal sinus osteomas are the frontal and ethmoidal sinuses [11,16]. The majority are diagnosed incidentally with a paranasal sinus CT scan. The most typical osteoma cases require only periodic imaging to follow their growth to prevent the development of complications. A paranasal sinus CT scan is a fundamental tool that not only permits the correct diagnosis but also supports the surgeon to plan the best surgical intervention.

The etiology of paranasal sinus osteomas is still being investigated. Various theories exist concerning the factors associated with their formation. Developmental, infective, and traumatic theories have been considered. Trauma and chronic sinusitis may stimulate osteoblast proliferation inside the sinus mucoperiosteum, causing tumor formation. The developmental theory is based on the fact that most osteomas arise at the junction of the ethmoid and frontal sinuses, where cartilaginous and membranous tissues meet during early embryonic life [9]. Our patient denied a history of trauma, but he had a history of chronic rhinosinusitis.

Surgical intervention is the treatment of choice for symptomatic giant ethmoid osteomas. However, the surgical options for such osteomas depend on multiple factors, including the size and location of the tumor, associated symptoms, presence of complications, experience and skills of the surgeon, and consent of the patient. A watchful waiting approach is preferred for small asymptomatic osteomas of the frontal and maxillary sinuses due to their relatively slow growth rate. On the contrary, masses of the ethmoid and sphenoid sinuses are recommended to be surgically removed despite their size or symptoms [12]. If an osteoma of the ethmoid sinus is small, and it is possible to extract it via the nostril, an endoscopic nasal approach by an otolaryngologist would be the best choice. Endoscopic intervention via the cavitation technique is usually preferred for the excision of an ethmoid sinus osteoma. However, an external approach, such as a lateral rhinotomy should be considered as an alternative [12,17], particularly in the presence of significant intraorbital extension.

Even though the mass was huge and there was minimal intraorbital extension in our case, we preferred to intervene endoscopically because the mass was accessible. We were able to remove the whole mass via the left nostril, and there was no need for an external approach.

## Conclusions

Giant osteomas of the ethmoid sinuses are rare and incidentally discovered on CT. They may cause serious symptoms due to intracranial or intraorbital extension. Headache and ocular signs are frequently reported symptoms. The gold standard treatment for giant osteomas is a surgical intervention, via an external approach or an endoscopic technique. The outcome of surgery for giant osteomas is excellent with rare recurrence.
